# Therapeutic mechanism and key active ingredients of Yinxing Mihuan Oral Solution in coronary heart disease comorbidity with anxiety: A network pharmacology and molecular docking approach

**DOI:** 10.1097/MD.0000000000040183

**Published:** 2024-10-25

**Authors:** Jiajun Jin, Huaigang Chen, Hong Wang, Yuncheng Gu, Liu Yang

**Affiliations:** a Department of Cardiology, Jiangxi Provincial People’s Hospital, The First Affiliated Hospital of Nanchang Medical College, Nanchang, Jiangxi Province, China; b Medical College of Nanchang University, Nanchang, Jiangxi Province, China; c Department of Science and Education, Jiangxi Provincial People’s Hospital, The First Affiliated Hospital of Nanchang Medical College, Nanchang, Jiangxi Province, China.

**Keywords:** active components, anxiety disorders, coronary heart disease, molecular mechanism, network pharmacology

## Abstract

Yinxing Mihuan Oral Solution (YMOS) is a Chinese patent medicine for treating coronary heart disease combined anxiety (CHDCA), but the molecular mechanism of its treatment is still unclear. This article aims to understand the molecular mechanism, optimize clinical drug use, and guide new drug development. Using the Swiss Target Prediction database, we obtained the main chemical composition of YMOS. Then we used network pharmacology to identify their potential targets. Network construction, coupled with protein–protein interaction and enrichment analysis was used to identify representative components and core targets. Finally, molecular docking simulation was conducted to further refine the drug–target interaction. Forty-two active chemicals were found in YMOS and 91 target genes related to CHDCA. The treatment effect was found to be associated with 1908 biological processes and 160 pathways, as revealed by the outcomes of the enrichment analysis. The potential therapeutic mechanisms of the drug are closely related to its antioxidant, anti-inflammatory, and vascular function regulation pathways, and the main core targets include albumin, tumor necrosis factor, TP53, AKT serine/threonine kinase 1, interleukin 1 beta, and vascular endothelial growth factor A. The potential molecular mechanisms of YMOS in CHDCA treatment were identified using network pharmacology and molecular docking approaches. The results reveal the systemic biological implications of YMOS. This study has systematically uncovered the molecular mechanism of YMOS for the first time, offering fresh insights for evidence-based clinical applications.

## 1. Introduction

Coronary heart disease (CHD) is a leading cause of death and disability worldwide, affecting millions of people and placing a huge burden on healthcare systems.^[1]^ The ailment is characterized by the narrowing or blockage of the coronary arteries responsible for supplying the heart muscle with oxygen and blood, leading to chest pains, shortness of breath, and increased vulnerability to heart attacks and sudden cardiac arrest.^[2]^ Although traditional risk factors such as smoking, hypertension, and hyperlipidemia have a considerable impact on the onset and advancement of coronary heart disease, it is widely recognized that psychosocial factors play a significant role in its development as well.

Moreover, these variables exert a substantial influence on the pathogenesis and prognosis of coronary heart disease. Anxiety disorders are the most prevalent mental afflictions among psychosocial factors, with an incidence rate of nearly 1 in 5 among adults in the United States.^[3]^ Previous studies have reported the positive correlation between anxiety disorders and increased risk of coronary heart disease and its complications.^[4,5]^ The standard treatment for anxiety in CHD patients involves pharmacological and non-pharmacological interventions.^[6]^ Nonetheless, the treatments available for coronary heart disease with anxiety have their own limitations and drawbacks, including adverse reactions, drug interactions, low compliance, high cost, and inadequate efficacy. Alternative and supplementary therapies are needed to provide more efficient and safer treatment options.^[7]^

The practice of Traditional Chinese Medicine (TCM) is a comprehensive and personalized approach to wellness and illness that has been utilized for numerous centuries in China and other regions of Asia. TCM utilizes a range of techniques for identifying and addressing medical conditions, such as herbal remedies, acupuncture, massage therapy, dietary modifications, and physical exercises. Yinxing Mihuan Oral Solution (YMOS) is a complex formulation that consists of various Chinese herbal medicines (the usage and dosage of it are oral, 10 mL each time), including ginkgo leaf extract and Armillariella Mellea Powders. In the realm of managing coronary heart disease and anxiety, it has exhibited favorable outcomes in mitigating symptoms, enhancing quality of life, and prolonging survival.^[8]^ However, the scientific evidence for TCM is still limited due to a lack of rigorous design, standardized interventions, objective outcome measures, and mechanistic interpretation.^[9,10]^ The precise mode of operation and essential constituents of YMOS remain obscure. Therefore, it is crucial to utilize modern scientific methodologies for exploring the therapeutic mechanism and essential components of YMOS in the treatment of coronary heart disease coupled with anxiety. The field of network pharmacology is founded upon the integration of several disciplines, including pharmacology, bioinformatics, genomics, proteomics, metabolomics, and systems biology. By analyzing the chemical structures, physicochemical properties, absorption, distribution, metabolism, excretion, and toxicity characteristics of TCM compounds, it becomes possible to identify potential targets, pathways, and mechanisms of action. In addition, a valuable approach is the utilization of molecular docking, which is a computational methodology that models the binding interaction between molecules based on their respective three-dimensional structures, electrostatic forces, hydrophobic interactions, and van der Waals forces. Molecular docking can evaluate the affinity, stability, and specificity of TCM compounds for targets based on their binding energy, conformation, and orientation. The present discourse aims to expound on the operational mode and fundamental active constituents of YMOS. We hypothesize that YMOS has the potential to ameliorate the pathophysiology of coronary heart disease accompanied by anxiety by means of the regulation of multiple molecular targets and signaling pathways. The aforementioned measures can serve to safeguard the myocardium, impede oxidative stress, enhance endothelial function, establish equilibrium within the autonomic nervous system, and effectively alleviate symptoms of anxiety and depression. In order to examine our hypothesis, we conducted an analysis utilizing network pharmacology and molecular docking techniques. Our investigation focused on the principal active constituents present in YMOS, with the aim of forecasting their potential targets and signaling pathways, and assessing their capacity for binding and stability to these targets. This study provides new scientific evidence and theoretical basis for using YMOS to treat coronary heart disease combined anxiety (CHDCA).

## 2. Materials and methods

### 2.1. Collection and screening of compound ingredients and disease genes

Utilize the Swiss Target Prediction database, available at http://swisstargetprediction.ch/, to obtain references for the pertinent constituents of the compound. Query the database to identify the targets associated with the components, and opt for the targets with a probability exceeding 0 as the targets of the components. Using “Anxiety” and “coronary heart disease” as keywords, disease-related genes were collected from the following databases: (1) DisGeNET database (https://www.disgenet.org/), with a screening criterion of score-gda >0.1; (2) Genecards database (https://www.genecards.org/), with a screening criterion of relevance score >10; and (3) OMIM database (https://omim.org/). The intersection of the genes from the 3 databases was taken to obtain the intersection gene.

### 2.2. Construction and analysis of medicinal material–ingredient–target–disease network and protein–protein interaction (PPI) network

We incorporated the ingredients, targets and intersection genes into Cytoscape 3.7.2. The Cytoscape software can be utilized to construct a network that links herbs, ingredients, targets, and diseases. Utilize the CytoNCA plug-in to conduct network topology analysis by importing the shared target dataset into the STRING database (https://string-db.org/). Select the species as *Homo sapiens*, screen the PPI data based on the confidence score provided by the STRING database, and establish a minimum confidence score threshold of 0.4. Download the “string_interactions_short.tsv” file and import it into Cytoscape 3.7.2 for network visualization. Finally, employ the CytoNCA plug-in to perform network topology analysis.

### 2.3. Gene Ontology (GO) and Kyoto Encyclopedia of Genes and Genomes (KEGG)

The utilization of R packages including “ClusterProfiler,” “org.Hs.e.g..Db,” and “ggplot2” in R programming language, enables the execution of GO and KEGG enrichment analysis, as well as core target visualization. In order to obtain gene annotation information, “org.Hs.e.g..Db” is employed. During the enrichment process, the adjusted *P*-value threshold is set at .05, while the q-value threshold is maintained at 0.05.

### 2.4. Molecular docking verification

Molecular docking analysis was used to calculate the affinity of the key hubs in the network. The ligands’ 3D structures were obtained from the PubChem database through downloading. The receptor’s three-dimensional configuration was acquired through utilization of the RCSB database, which can be found at http://www.rcsb.org/. The ligands and receptors were repaired using the mgltools_win32_1.5.6 software and saved as PDBQT files. The assessment of docking affinity between the target proteins and the key active ingredients was performed using the Auto Dock Vina 1.1.2 software, which can be accessed through http://vina.scripps.edu/. The ultimate graphical representation of the molecular docking information was achieved through the utilization of Discovery Studio 2016.

## 3. Results

### 3.1. Collection and screening of compound ingredients and disease genes

**Figure 1.**
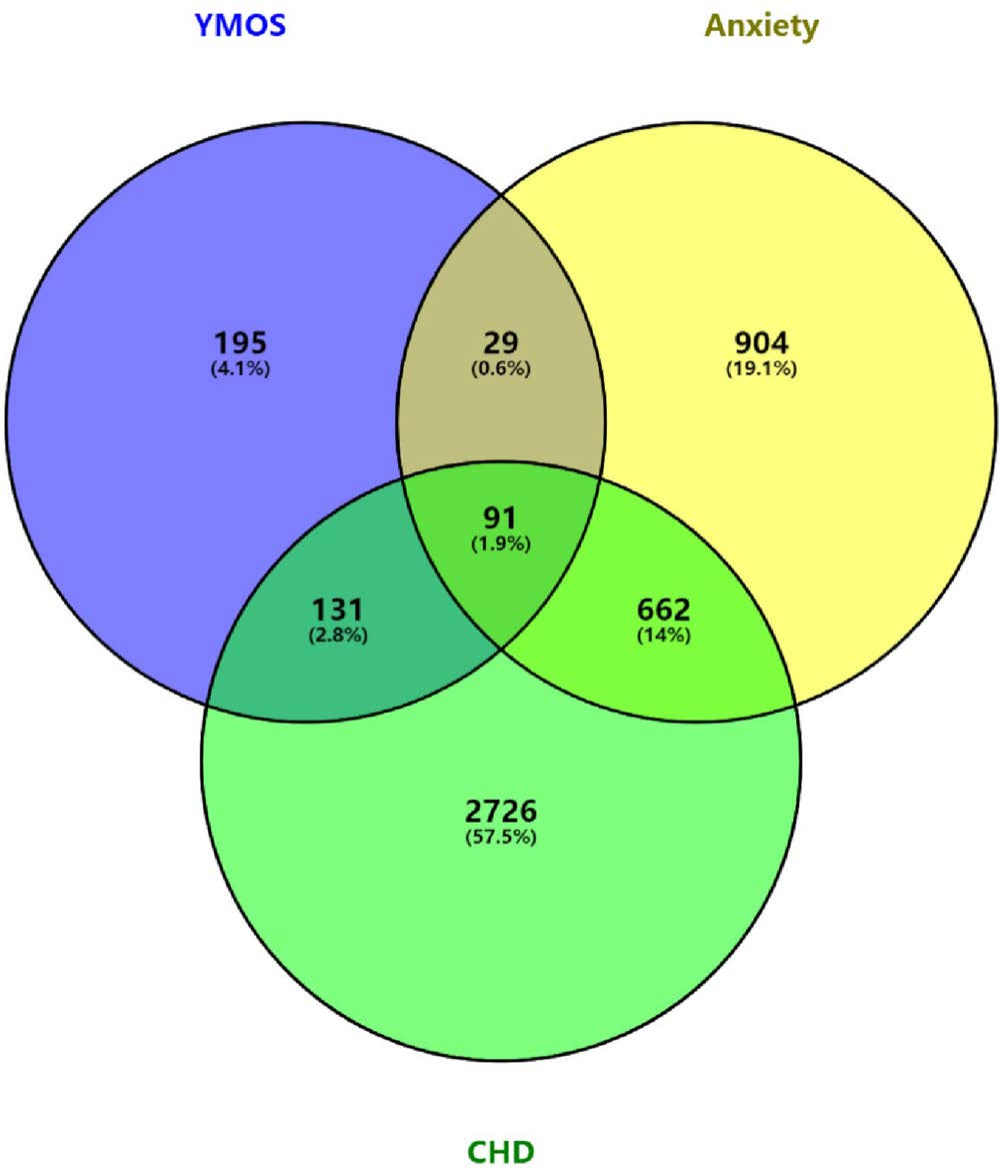
Screening of common targets and active ingredients of YX-CADCA and the Venn diagram of common targets. CHD, coronary artery heart disease; YMOS, Yinxing Mihuan Oral Solution.

We determined that YMOS comprises 42 distinct components after a thorough literature investigation. By searching the Swiss Target Prediction database and filtering out targets with a probability less than or equal to 0, we found 446 targets present in YMOS. A total of 3610 coronary heart disease targets, 1686 anxiety targets, and 91 intersecting genes were obtained from the database (Fig. 1). While the prime treatment objectives of YMOS for CHDCA may revolve around these 91 targets.

### 3.2. Construction and analysis of medicinal material–ingredient–target–disease network and PPI network

**Figure 2.**
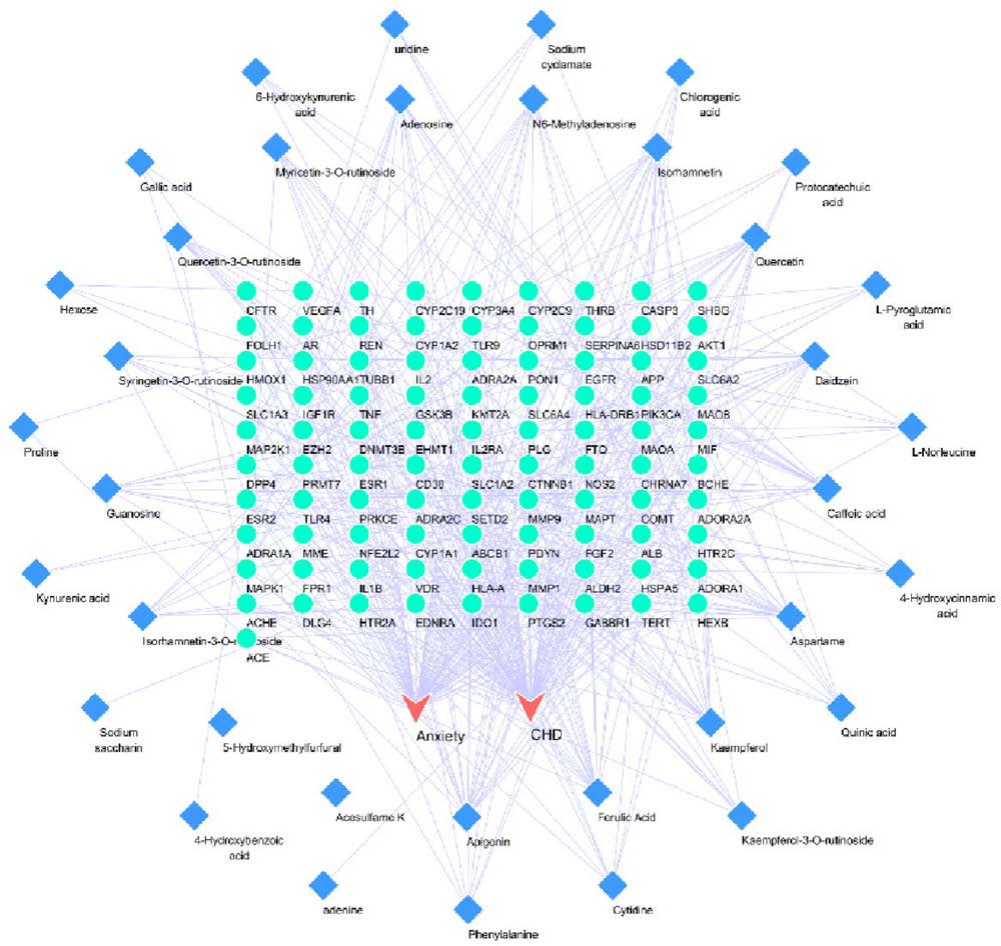
Herb-ingredient–target-disease network. The diamond-shaped nodes in the diagram represent the active components of YM, which consist of 42 ingredients. Meanwhile, the circular nodes denote the common targets of these components, amounting to a total of 91 targets.

**Figure 3.**
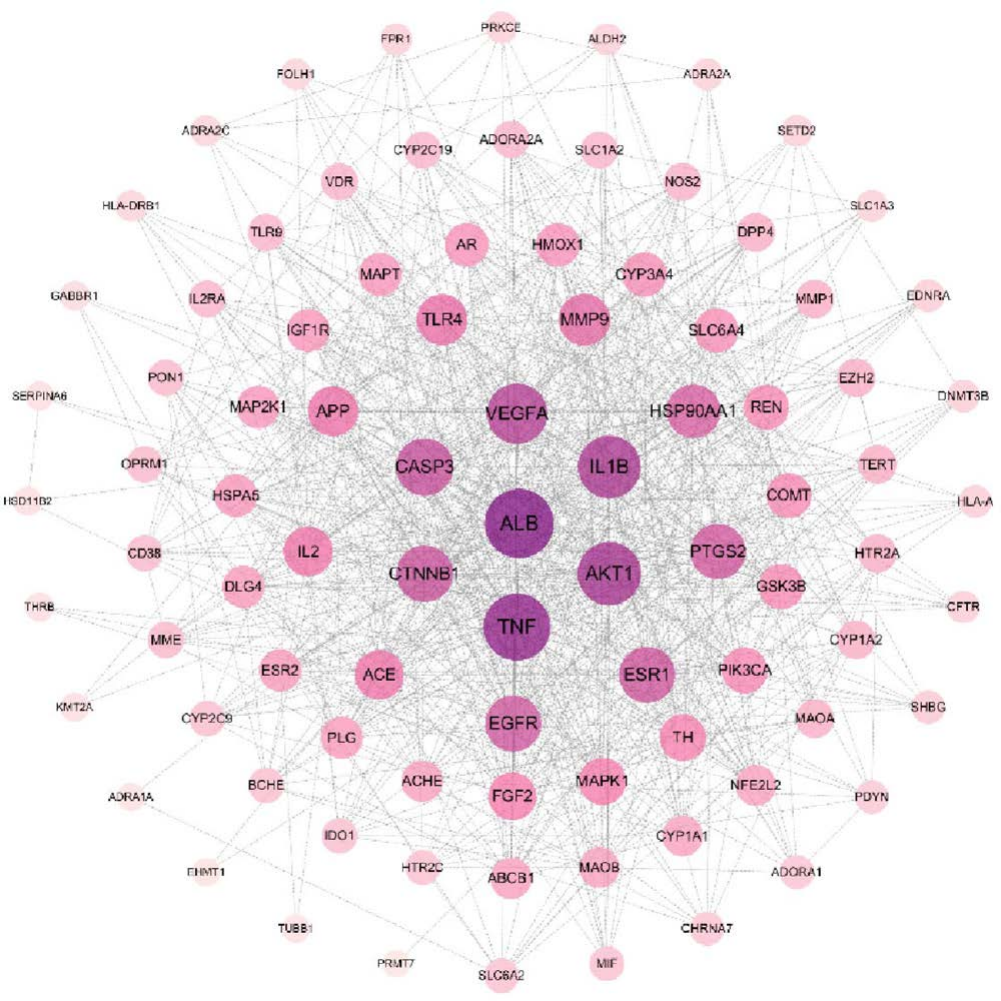
PPI network of common goals. The protein nodes are arranged in descending order based on their degree value and are shaded in a gradient ranging from deep purple (representing high values) to pale lavender (representing low values). The median degree value is determined to be 5.

The current software and network infrastructure have generated a network that maps out the relationships between medicinal materials, their corresponding ingredients, targeted diseases, and associated effects. This network comprises a total of 129 nodes and 480 edges. The most prominent components, as determined by degree value, include ferulic acid, apigenin, kaempferol, daidzein, and caffeic acid, among others (see Fig. 2). Import the common target dataset into the STRING database to obtain the protein interaction network. Then, import the TSV file of the PPI network into Cytoscape, which includes 89 related targets and 935 interrelationships between targets (refer Fig. 3). The protein nodes have been categorized based on their degree values, with the darker purple nodes representing higher values and the lighter lilac nodes representing lower values. The neutral color, purple, has been assigned to the median degree of 5 in the network, which comprises 89 nodes and 935 edges. The core targets of this network include albumin (ALB), tumor necrosis factor (TNF), TP53, AKT serine/threonine kinase 1 (AKT1), interleukin 1 beta (IL1B), vascular endothelial growth factor A (VEGFA), and others.

### 3.3. GO and KEGG enrichment

**Figure 4.**
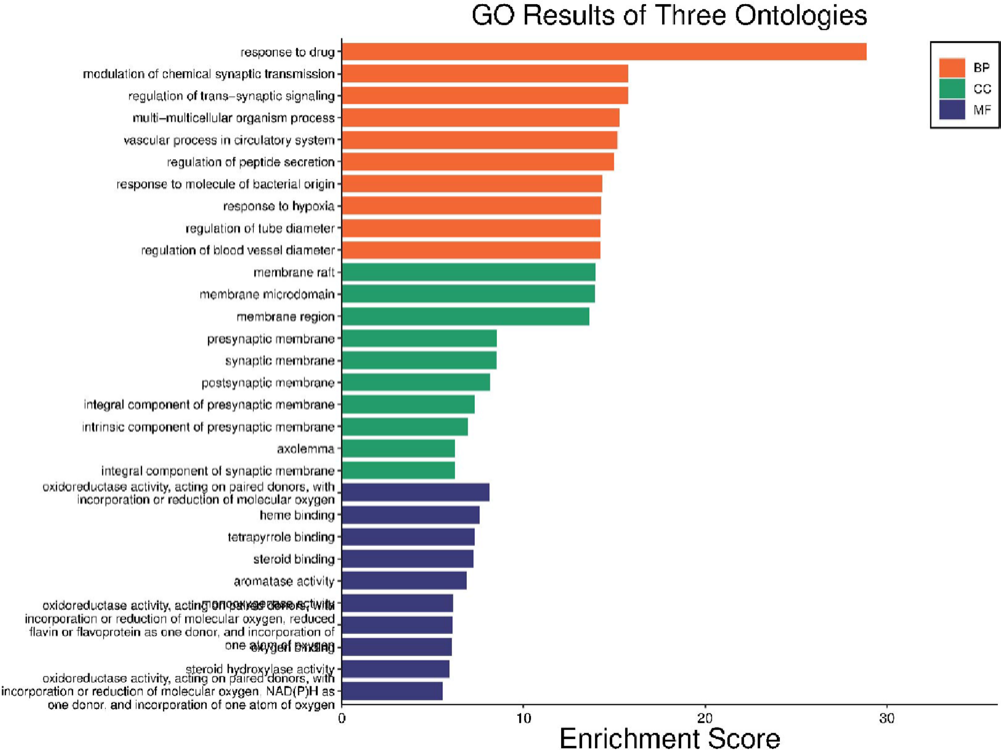
The results of GO enrichment analysis. Including GO biological processes (orange), GO cellular components (green), GO molecular functions (blue).

**Figure 5.**
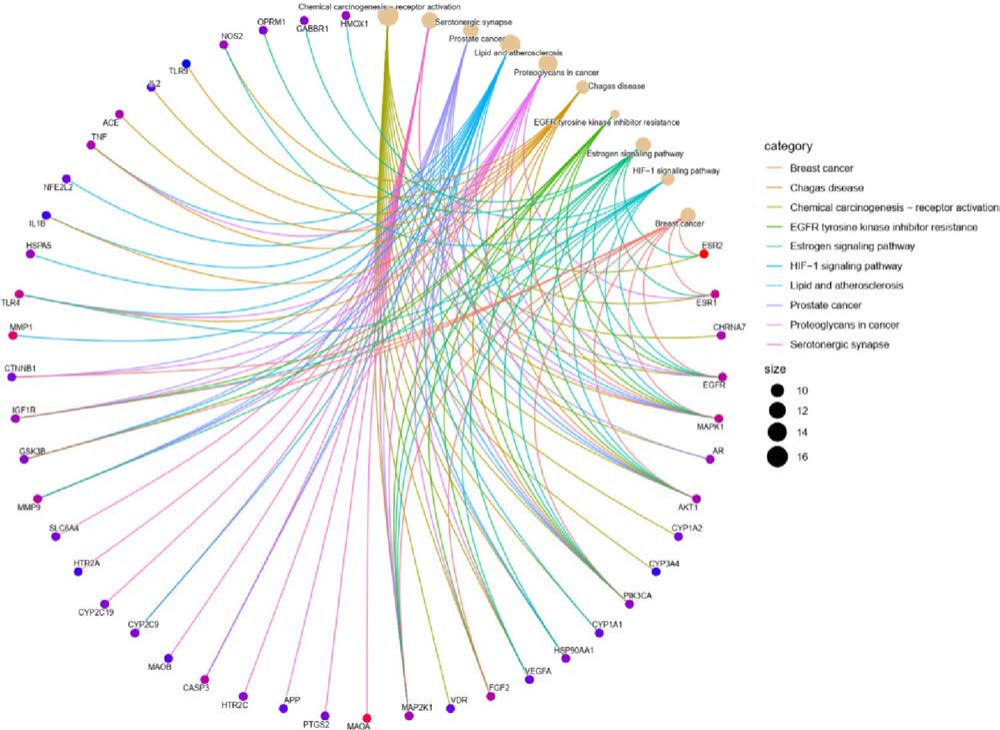
The significant items from the results of KEGG enrichment analysis are listed in the upper right corner of the figure.

Upon conducting GO enrichment analysis, it was discovered that a total of 1908 biological processes (BP) were enriched. These processes encompassed a diverse range of functions, including response to drugs, regulation of chemical synaptic transmission, regulation of transsynaptic signaling, as well as various multicellular and vascular processes within the circulatory system. The circulatory system is characterized by a range of vascular processes, including but not limited to vascular smooth muscle contraction, migration, proliferation, angiogenesis, and remodeling. The nervous system is potentially involved in drug response, the regulation of chemical synaptic transmission, and the regulation of transsynaptic signal transduction. These processes may have a bearing on the treatment or mitigation of anxiety through the administration of YinXin MinHuan Oral Solution. The identified molecular functions (MFs) are primarily involved in essential cellular processes such as metabolism, oxygen transport, hormone regulation, and estrogen synthesis. Such MFs are instrumental in highlighting the influence of differentially expressed genes on cell metabolism and endocrine functions. An in-depth analysis of KEGG pathway enrichment revealed 160 crucial pathways, including noteworthy entries such as chemical carcinogenesis–receptor activation, serotonergic synapse, prostate cancer, lipid and atherosclerosis, proteoglycans in cancer, and others (as shown in Fig. 5). The cellular components (CCs) were significantly enriched with differentially expressed genes. These CCs mainly involve in cell membrane and neurotransmission, which may indicate effects of differentially expressed genes on the structure and function of the cell membrane, and modulating neuronal signal transduction. In addition, a total of 182 MFs were discovered, encompassing a diverse range of activities such as oxidoreductase activity, heme binding, tetrapyrrole binding, steroid binding, aromatase activity, and many others. The results are presented in Figure 4, which illustrates the identified MFs that are primarily associated with critical cellular processes such as metabolism, oxygen transport, hormone regulation, and estrogen synthesis. The aforementioned MFs provide valuable insights into the potential impacts of genes that are differentially expressed on both cell metabolism and endocrine function. Through KEGG pathway enrichment analysis, it was determined that there are 160 significant pathways, including entries such as chemical carcinogenesis–receptor activation, serotonergic synapse, prostate cancer, lipid and atherosclerosis, and proteoglycans in cancer, among others (as depicted in Fig. 5). Based on the results of enrichment analysis, we propose that YMOS can target multiple functions and biological factors of CHDCA, but its influence and extent still require further investigation and validation.

### 3.4. Molecular docking verification

**Figure 6.**
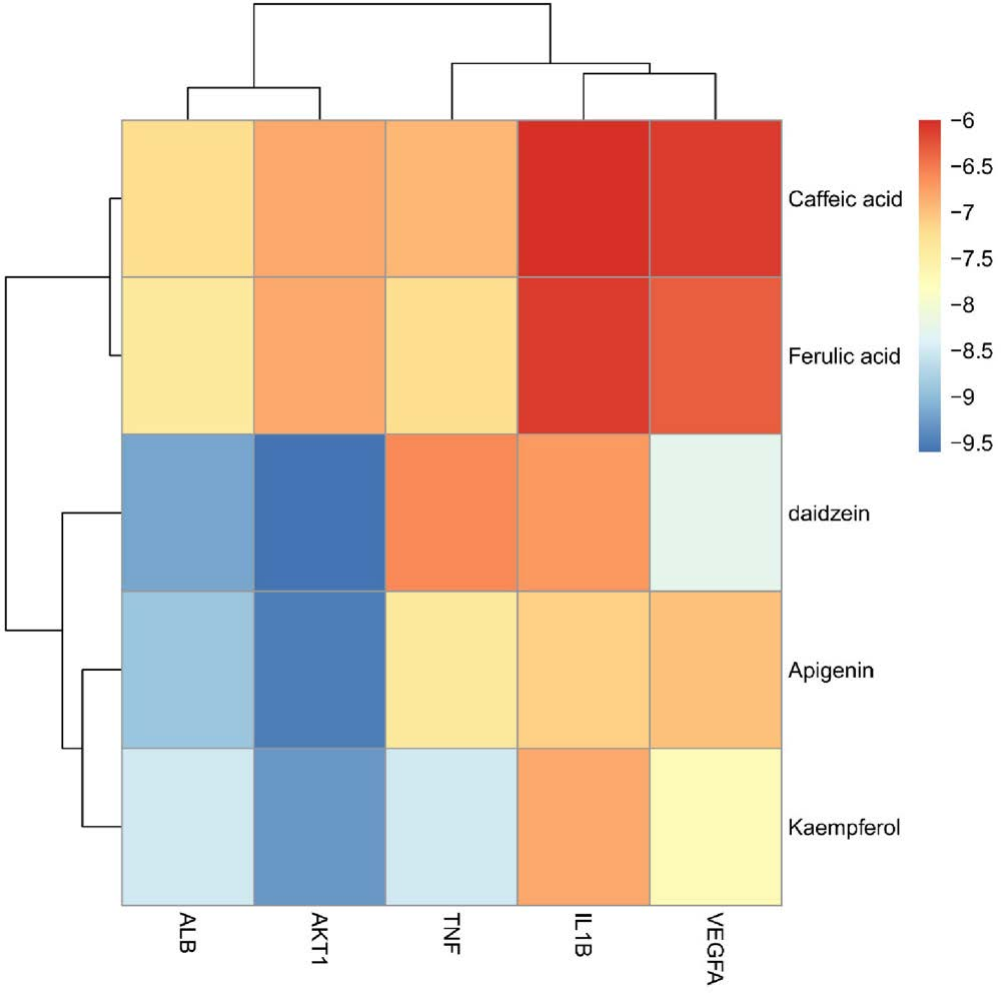
Key targets of herbal medicines and binding energy (kcal/mol) heat map of specific active ingredients.

**Figure 7.**
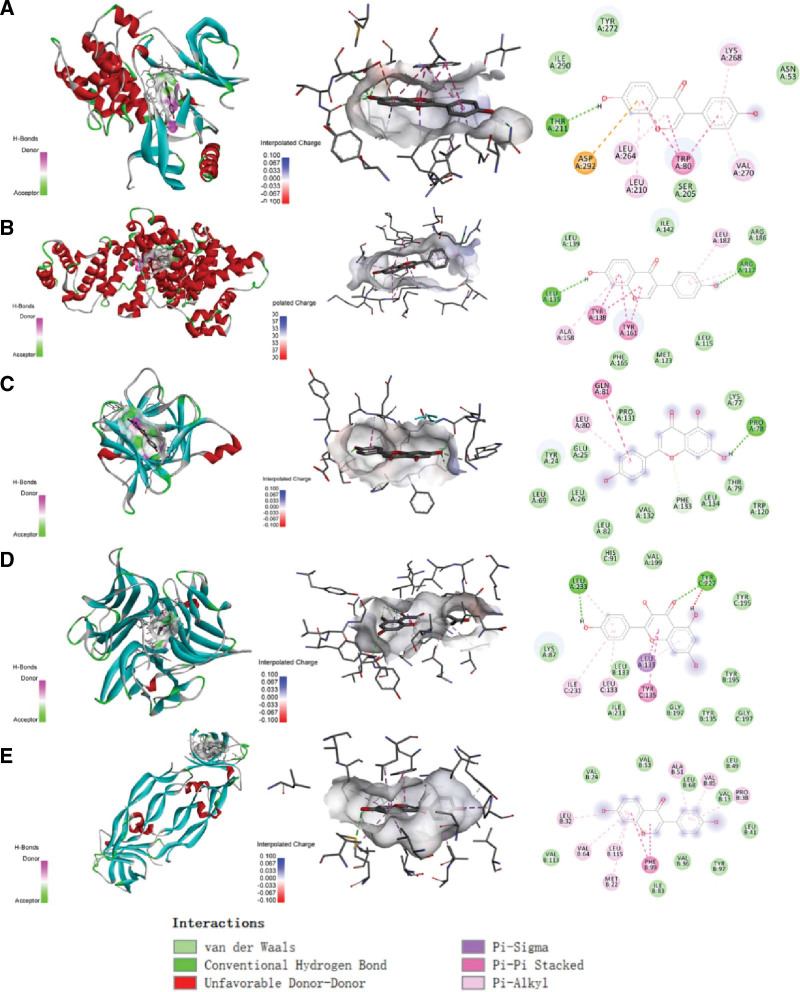
Molecular docking visualization. (A) AKT1 and daidzein binding pattern; (B) ALB and daidzein binding pattern; (C) IL1B and apigenin binding pattern; (D) TNF and kaempferol binding pattern; (E) VEGFA and daidzein binding pattern.

To verify the affinity between the key components and the target, we performed molecular docking analysis. The binding ability between the target and the component was quantified by the binding energy (kcal/mol). A lower binding energy indicated a higher binding ability. The best docking results were visualized. The results demonstrated that ALB had a high affinity to each component, with a binding energy lower than −7 kcal/mol for each component. IL1B had a low affinity, with only 1 component having a binding energy lower than −7 kcal/mol. Daidzein showed the highest affinity to AKT1, with a binding energy of −9.6 (see Table 1 and Fig. 6). The molecular docking visualization results revealed that the compounds were all bound in the protein pocket and interacted with the amino acid residues in the pocket through hydrophobic forces, hydrogen bonds, P–π conjugation, and other mechanisms (see Fig. 7).

**Table 1:** Key targets of herbal medicines and binding energy (kcal/mol) of specific active ingredients.

Name	ALB	TNF	AKT1	IL1B	VEGFA
Apigenin	-8.9	-7.4	-9.5	-7.1	-7
Caffeic acid	-7.2	-6.9	-6.8	-6	-6.1
Daidzein	-9.2	-6.6	-9.6	-6.7	-8.3
Ferulic acid	-7.4	-7.2	-6.8	-6.1	-6.3
Kaempferol	-8.5	-8.5	-9.3	-6.8	-7.7

AKT1 = AKT serine/threonine kinase 1, ALB = albumin, IL1B = interleukin 1 beta, TNF = tumor necrosis factor, VEGFA = vascular endothelial growth factor A.

## 4. Discussion

CHD is one of the major causes of mortality and morbidity worldwide and is often complicated by anxiety disorders. Anxiety disorders worsen the prognosis and quality of life of CHD patients and increase the risk of cardiac events and death. The existing treatments for CHDCA are not sufficiently effective. Therefore, in addition to conventional cardiovascular drugs, CHDCA patients need new drugs and treatment modalities. The aim of this study was to investigate the therapeutic mechanism and key active ingredients of YMOS in coronary heart disease complicated with anxiety by using network pharmacology and molecular docking methods. The components and targets of YMOS were collected from the literature and the genes related to coronary heart disease and anxiety were collected from the database. A network of herbal medicine–component–target–disease was constructed and protein interaction network, GO and KEGG enrichment analyses, and molecular docking validation were performed. The results suggested that key proteins involved in multiple BP and signaling pathways related to CHDCA could be targeted by the active ingredients of YMOS, but further clarification by animal experiments and clinical trials was needed for the functional changes of these factors and the profound impact of treatment. The compound components and targets of YMOS were collected and it was found that YMOS contained 42 components and 446 targets. Subsequent target analysis revealed that 91 overlapping genes with CHDCA were present. A drug-herb–ingredient–target–disease network was constructed, which had a total of 129 nodes and 480 edges. The key components were ranked according to the degree value as follows: ferulic acid, apigenin, kaempferol, daidzein, caffeic acid. The TEPPI network results showed that the common targets of YMOS–CHDCA were mainly key proteins in the pathway. A strong affinity with multiple components was shown by ALB, TNF, TP53, AKT1, IL1B, VEGFA, and others according to the clustering coefficient. Inflammation, apoptosis, angiogenesis, and other processes might be regulated by these components and targets,^[11–13]^ which might have a therapeutic effect on CHDCA. Previous studies have performed qualitative and quantitative analyses on the quality assessment and stability studies of YMOS, and have identified 67 chemical components, of which 34 were confirmed by reference substances. This differs slightly from our results, which may be attributed to different analytical methods or batches.^[14]^ The active ingredients include various polyphenol compounds, which are abundant in plant-derived foods and exhibit anti-oxidative stress, anti-inflammatory and vascular regulatory effects.^[15]^ Among the polyphenolic compounds, ferulic acid is the most abundant. Previous literature has reported the synthesis and antioxidant activity of the sulfate derivatives of ferulic acid and caffeic acid and the acyl glucoside of ferulic acid. It was found that the sulfate derivatives of ferulic acid and caffeic acid had very low antioxidant activity, while the acyl glucoside of ferulic acid had strong antioxidant activity.^[16]^ Moreover, ferulic acid shares structural similarities or metabolic associations with other key components such as caffeic acid, apigenin, and kaempferol. Apigenin, kaempferol, daidzein, etc are also important polyphenol components, which are abundant in plant-derived foods and have similar biological activities. It has been reported that 6 phytochemical components, including apigenin, kaempferol, daidzein, etc, were isolated from asparagus root extract and evaluated for their effects on H_2_O_2_-induced oxidative stress. The results showed that asparagus root extract could significantly attenuate H_2_O_2_-induced cytotoxicity and reactive oxygen species production, and enhance intracellular glutathione levels and superoxide dismutase activity. These are all related to oxidative stress.^[17]^ Antioxidant, anti-inflammatory, and vascular regulatory actions may be beneficial for coronary heart disease and anxiety, as they can reduce oxidative stress and inflammation, protect vascular endothelial cells, improve blood perfusion, and lower the risk of myocardial ischemia and injury.^[18]^ Moreover, these effects can improve the function of neurons and neurotransmitters, and alleviate the emotional and behavioral symptoms of anxiety. Specifically, the mechanisms of anti-oxidation and anti-inflammation on mood may involve the following aspects: (1) protecting neurons from oxidative stress and inflammatory responses, and maintaining the integrity of neuronal structure and function^[19]^; (2) regulating the synthesis and release of neurotransmitters, such as dopamine, serotonin, norepinephrine, etc, and improving emotional state^[20]^; (3) affecting neural development and plasticity, promoting neuronal differentiation and synapse formation, enhancing learning and memory; (4) regulate the balance of neuroendocrine system and immune system, reduce the level of stress hormones and inflammatory factors, reduce stress.

GO enrichment analysis is a bioinformatics method that analyzes gene expression or protein expression data, and helps to identify the enrichment of genes or proteins in terms of CCs, molecular functions and BP. We found 1908 BP, such as responses to drugs, regulation of chemical synaptic transmission, regulation of transsynaptic signaling, multicellular BP, vascular processes in the circulatory system, etc; 103 CCs, such as membrane rafts, membrane microdomains, membrane domains, presynaptic membranes, synaptic membranes, etc; and 182 molecular functions, such as oxidoreductase activity, acting on paired donors, incorporation or reduction of molecular oxygen, heme binding, tetrapyrrole binding, steroid binding, aromatase activity, etc (Fig. 4). Moreover, based on the results of GO enrichment analysis, we inferred that the active components in YMOS mainly target the regulation of oxidative stress, which was discussed in the previous section. These are known to play an important role in the pathogenesis of CHDCA. Additionally, some of the 103 CCs and 182 molecular functions may be associated with coronary heart disease or anxiety, such as, oxidoreductase activity, response to oxidative stress, etc. These involve the intracellular production and clearance of free radicals, which are chemicals with unstable electrons that can cause cellular damage and death. Patients with coronary heart disease often have increased levels of oxidative stress, which may lead to cardiomyocyte injury and enhance the risk of myocardial ischemia and hypoxia.^[21,22]^ Patients with anxiety may also have increased levels of oxidative stress, which may cause disturbances in neurotransmitter metabolism and affect nervous system function.^[1,23,24]^ Thus, YMOS may modulate the level of oxidative stress by affecting the expression and activity of oxidoreductases, and thereby influence the onset and progression of coronary heart disease and anxiety. Heme binding and tetrapyrrole binding refer to the ability of some proteins to bind with iron-containing cyclic compounds such as heme or tetrapyrrole and participate in the transport and utilization of oxygen. Moreover, due to myocardial ischemia and hypoxia, patients with coronary heart disease require enhanced transport and utilization efficiency of oxygen in the blood, and hemopexin and tetrapyrrole binding proteins may play an important role in this process. Similarly, anxious patients also need to enhance the transport and utilization efficiency of oxygen in the blood due to emotional stress, and the interaction of hemopexin and tetrapyrrole may influence this. The subsequent KEGG pathway enrichment analysis identified 160 significant pathways, and the top entries included chemical carcinogenesis–receptor activation, serotonergic synapse, prostate cancer, lipid and atherosclerosis, Proteoglycans in cancer, etc (Fig. 5). Based on the results of enrichment analysis, we suggest that YMOS can target multiple functions and biological factors of CHDCA, but its impact and extent still require further investigation and validation. Among them, the chemical carcinogenesis–receptor activation pathway (No. hsa05204) involves the damage and transformation of cells by chemical substances, which may be associated with the development of coronary heart disease, because oxidative stress and inflammation can also cause vascular endothelial cell injury and atherosclerosis.^[25]^ Anxiety may increase the exposure to chemical carcinogens, such as smoking, drinking, etc, and thereby exacerbate the risk of coronary heart disease.^[26,27]^ Moreover, other entries, such as serotonergic synapse, prostate cancer, lipid and atherosclerosis, proteoglycans in cancer, etc, are also relevant. Among them, the serotonergic synapse pathway involves the synthesis, release and signal transduction of serotonin (5-HT). 5-HT is an important neurotransmitter that regulates physiological functions such as mood, sleep and appetite. 5-HT also plays an important role in coronary heart disease, as it can affect platelet aggregation, vasoconstriction and smooth muscle proliferation and other processes, and thereby influence the stability and blood perfusion of coronary arteries.^[28]^ Anxiety may cause disturbances in the 5-HT system, and thereby affect cardiovascular function. Other pathways, such as prostate cancer, are involved in the pathogenesis of prostate cancer, mainly related to the androgen receptor (AR) signaling pathway. AR signaling pathway also plays a role in coronary heart disease. It can regulate the proliferation, migration and apoptosis of vascular smooth muscle cells, and thereby influence the formation and progression of atherosclerosis.^[29]^ Anxiety may affect the level of androgen and the activity of the AR signaling pathway, and thereby influence the incidence and prognosis of coronary heart disease.^[30]^ The lipid and atherosclerosis pathway is involved in lipid metabolism and the formation of atherosclerosis, mainly related to cholesterol, triglyceride, apolipoprotein, and other factors. These factors play an important role in coronary heart disease, as they can affect the integrity of the vessel wall, endothelial function, inflammatory response and other processes, and thereby affect the obstruction and ischemia of coronary arteries. Anxiety may affect lipid metabolism and atherosclerotic processes through mechanisms such as activation of the sympathetic nervous system, induction of oxidative stress, and increased cortisol secretion.^[1]^ Proteoglycans in cancer pathway involves the role of proteoglycans in cancer, mainly related to cell adhesion, migration, proliferation, apoptosis, and other processes. Proteoglycans also play a role in coronary heart disease. They can regulate the structure, elasticity, and permeability of vessel walls, and thereby influence hemodynamics and the formation and progression of atherosclerosis.^[1,31]^ Anxiety may affect the synthesis and degradation of proteoglycans, and thereby affect the characteristics and functions of vessel walls.

Moreover, we performed molecular docking on the screened active ingredients and key targets to validate the results of network pharmacology. Since it is a virtual screening method, molecular docking may involve some real-world biases that may cause some discrepancies in in vivo/in vitro experiments. However, docking results reflect potential therapeutic mechanisms and provide guidance for animal validation experiments. Currently, network pharmacology analyzes multiple components and molecular docking technology is used to verify the affinity between key components and targets. The binding ability between targets and components is measured by binding energy (kcal/mol). The results showed that ALB had a good binding ability to each component, and the binding energy to each component was less than −7 kcal/mol. IL1B was poor, and only 1 component had a binding energy less than −7 kcal/mol. The binding energy of daidzein to AKT1 was the best, with a binding energy of −9.6 (see Table 1 and Fig. 6). The molecular docking visualization results showed that the compounds were all bound in the protein pocket, and formed hydrophobic forces, hydrogen bonds, and P–π conjugation with the amino acid residues in the pocket.

Furthermore, for various key genes, we have further explored their specific mechanisms of action in cardiovascular diseases and anxiety disorders, as well as the biological significance of their interactions with YMOS components. For instance, ALB not only plays a pivotal role in regulating plasma osmolarity, but it may also alleviate the severity of cardiovascular diseases by inhibiting inflammatory responses.^[32]^ We found that ALB exhibits good binding affinity with multiple components in YMOS, which may contribute to its potential application in clinically mitigating cardiovascular pathologies. TNF, a major inflammatory and immune mediator, has been extensively studied for its role in cardiovascular diseases and anxiety disorders. The interaction between TNF and YMOS components in our study further validates the possibility of achieving therapeutic effects by modulating these inflammatory pathways.^[33]^ AKT1, an important protein in cellular survival signaling pathways, has its activity directly related to cardiovascular health.^[34]^ The high affinity of YMOS components to AKT1 suggests that these components may exert a protective effect on the cardiovascular system by influencing AKT1’s activity.

We also noticed that, although the binding capacity of IL1B is relatively weak, its role in regulating inflammatory responses cannot be overlooked.^[35]^ Further research is needed to investigate how YMOS components influence IL1B-related signaling pathways, in order to discover new therapeutic mechanisms for diseases. VEGFA, a crucial regulator of angiogenesis and repair, has potential significance in improving cardiovascular health through YMOS’s actions on it.^[36]^ By exploring these interactions and mechanisms, we can gain a deeper understanding of the comprehensive efficacy of YMOS and its potential clinical applications.

Our results are somewhat innovative and meaningful. First, we used network pharmacology and molecular docking methods to reveal the therapeutic mechanism and key active components of YMOS in CHDCA from the holistic and molecular levels, providing a theoretical basis for its clinical application. Second, we identified 91 common genes shared by YMOS and CHDCA, providing evidence for its characteristic of treating different diseases with the same treatment.

## 5. Limitation

The limitation of this study is mainly the lack of experimental further validation. Apart from this, since it is a virtual screening method, molecular docking may involve some real-world biases that may cause some discrepancies in in vivo/in vitro experiments. “Secondly, it is currently difficult to fully elucidate the compatibility rules and the weight of its effects. In the future, we will need to rely on technologies such as componentomics and metabolomics to conduct in-depth analysis of the synergistic enhancement and antagonistic restriction mechanisms of the compound prescription.” Furthermore, while the study focuses on the pharmacological mechanism, it pays insufficient attention to the potential toxic and side effects of Yinxing Mihuan Oral Solution. Given that this oral liquid contains complex chemical components, some of which may possess toxic effects, systematic toxicological research is still needed in the future to evaluate its toxicity impact on major organs and systems, define the safe dosage range, and formulate reasonable drug administration plans and precautions. This is intended to provide a more comprehensive basis for clinical rational drug use.

## 6. Conclusion

In this study, we found that the potential therapeutic mechanism of the drug is closely related to the mechanisms of anti-oxidation, anti-inflammation, and vascular function regulation, as well as the processes of oxidoreductase activity and oxidative stress. These BP are influenced by chemical carcinogenesis–receptor activation, serotonergic synapse, prostate cancer, lipid and atherosclerosis, proteoglycans in cancer, and other pathways. Molecular docking results showed that the active components of YMOS and key proteins, namely ALB and apigenin, caffeic acid, daidzein, ferulic acid, kaempferol have stable interactions in the context of coronary heart disease complicated with anxiety. Our study reveals the systemic biological implications of YMOS. Future studies could aim to develop potential applications of YMOS or to confirm our findings.

## Author contributions

**Conceptualization:** Jiajun Jin, Huaigang Chen, Liu Yang.

**Funding acquisition:** Hong Wang, Liu Yang.

**Methodology:** Jiajun Jin, Huaigang Chen, Liu Yang.

**Project administration:** Yuncheng Gu.

**Software:** Huaigang Chen, Yuncheng Gu.

**Supervision:** Hong Wang, Yuncheng Gu, Liu Yang.

**Visualization:** Huaigang Chen.

**Writing – original draft:** Jiajun Jin.

**Writing – review & editing:** Hong Wang, Liu Yang.
